# Circulating endocannabinoids and cognitive function in older adults

**DOI:** 10.1002/alz70856_105642

**Published:** 2026-01-09

**Authors:** Shiraz Vered, Alexa S Beiser, Liron Sulimani, Sharon Sznitman, Saptaparni Ghosh, Gil M Lewitus, David Meiri, Sudha Seshadri, Galit Weinstein

**Affiliations:** ^1^ School of Public Health, University of Haifa, Haifa, Mount Carmel, Israel; ^2^ Boston University School of Public Health, Boston, MA, USA; ^3^ Boston University School of Medicine, Boston, MA, USA; ^4^ The Framingham Heart Study, Framingham, MA, USA; ^5^ Boston University Chobanian & Avedisian School of Medicine, Boston, MA, USA; ^6^ Department of Biostatistics, Boston University School of Public Health, Boston, MA, USA; ^7^ Department of Neurology, Boston University Chobanian & Avedisian School of Medicine, Boston, MA, USA; ^8^ Technion‐Israel Institute of Technology, Haifa, Israel; ^9^ University of Haifa, Haifa, Israel; ^10^ Glenn Biggs Institute for Alzheimer's & Neurodegenerative Diseases, University of Texas Health Science Center, San Antonio, TX, USA; ^11^ Department of Neurology, Boston University School of Medicine, Boston, MA, USA; ^12^ Framingham Heart Study, Framingham, Boston, MA, USA; ^13^ Department of Neurology, University of Texas Health Sciences Center, San Antonio, TX, USA, San Antonio, TX, USA; ^14^ University of Texas Health San Antonio, San Antonio, TX, USA

## Abstract

**Background:**

Endogenous cannabinoids (endocannabinoids; eCBs) are small lipophilic signaling molecules implicated in multiple functions including neuromodulation and neuroinflammation. While the role of eCBs in cognitive‐related processes has been demonstrated in preclinical studies, observational studies are limited, particularly by exploration of only few ‘classical’ eCB compounds. We assessed the relationship of multiple circulating eCBs and eCB‐like molecules with cognitive function, and tested for effect‐modification by sex and apolipoprotein ɛ4 (ApoEɛ4) genotype in a sample of dementia‐free older adults.

**Method:**

In this cross‐sectional study, serum levels of 44 eCBs were analyzed in cognitively healthy participants aged ≥65 years of the Framingham Heart Study Offspring cohort who attended examination cycle 9 (2011‐2014). Linear regression models were used to examine the associations of eCB serum levels with cognitive function while adjusting for potential confounders including age, sex, education, ApoEɛ4, obesity and time between the eCB measurement and cognitive assessment. Effect modification by sex and ApoEɛ4 was additionally examined by including interactions terms in the models and stratification.

**Result:**

A total of 237 participants were included (mean age 73.3±6.2 years and 95 (40%) men). After correction for multiple comparisons, increased levels of *linoleic acid*, *linolenic acid*, *oleic acid*, *oleoyl alanine* and *palmitoyl alanine* were associated with poorer executive function (B±SE=‐0.0002±0.0001, *p* = 0.002; B±SE=‐0.0005±0.0001, *p* <0.001; B±SE=‐0.0002±0.0001, *p* = 0.003; B±SE=‐0.74±0.25, *p* = 0.003 and B±SE=‐1.75±0.62, *p* = 0.005, respectively). In addition, elevated levels of *linolenoyl amide* and *linoleoyl amide* were associated with poorer verbal memory (B±SE=‐1.45±0.44, *p* = 0.001 and B±SE=‐0.16±0.05, *p* <0.001, respectively) and attention (B±SE=‐0.12±0.04, *p* <0.001 and B±SE=‐0.013±0.004, *p* <0.001, respectively). A significant interaction with sex was observed such that most of the associations were present only among women. Furthermore, associations between several eCBs and perceptual organization were observed only among participants with ApoEɛ4 genotype.

**Conclusion:**

We identified novel eCBs compounds that may be related to cognitive function among dementia‐free older adults. Additionally, we demonstrated wide range of sex‐specific and ApoEɛ4‐specific associations between eCBs and cognitive function. The variation in the direction of the eCB‐cognition associations, together with the evidence of sex and ApoEɛ4 genotype, highlight the complexity of the eCBome and the need for validations of our findings in additional samples.

